# Overexpression of Eukaryotic Translation Initiation Factor 5A2 (EIF5A2) Correlates with Cell Aggressiveness and Poor Survival in Gastric Cancer

**DOI:** 10.1371/journal.pone.0119229

**Published:** 2015-03-20

**Authors:** Qing-Bin Meng, Wei-Ming Kang, Jian-Chun Yu, Yu-Qin Liu, Zhi-Qiang Ma, Li Zhou, Quan-Cai Cui, Wei-Xun Zhou

**Affiliations:** 1 Department of General Surgery, Peking Union Medical College Hospital, Chinese Academy of Medical Science and Peking Union Medical College, Beijing, China; 2 Department of Gastrointestinal Surgery, the First Hospital of Wu Han City, Wuhan city, Hubei Provence, China; 3 Cell Center, Institute of Basic Medical Sciences, Chinese Academy of Medical Sciences, Wuhan city, Beijing, China; 4 Department of Pathology, Peking Union Medical College Hospital, Chinese Academy of Medical Science and Peking Union Medical College, Beijing, China; Sun Yat-sen University Medical School, CHINA

## Abstract

Eukaryotic translation initiation factor 5A2 (EIF5A2) plays an important role in tumor progression and prognosis evaluation. However, little information is available about its potential role in gastric cancer. This study aimed to investigate the function of EIF5A2 in tumor progression and its potential mechanisms. EIF5A2 expression was measured in human gastric cancer cell lines, the immortalized gastric mucosal epithelial cell line (GES-1) and human gastric cancer tissues and knocked down by RNA interference or upregulated by EIF5A2 plasmid transfection. Cell proliferation, migration and invasion were assessed *in vitro*. The downstream targets of EIF5A2 were examined by western blotting. EIF5A2 and its potential target metastasis-associated protein 1 (MTA1) expression were examined in 160 pairs of human gastric cancer and adjacent non-tumor specimens using immunohistochemistry (IHC) staining, and its correlation with clinicopathological features and survival was investigated. Knockdown of *EIF5A2* or *MTA1* caused an apparent suppression of HGC27 cell proliferation, migration and invasion. After knockdown of *EIF5A2* in HGC27 cells, E-cadherin levels were upregulated and vimentin, cyclin D1, cyclin D3, C-MYC and MTA1 levels were downregulated. Upregulation of EIF5A2 in MKN45 cells resulted in the converse. IHC results showed a positive correlation between EIF5A2 and MTA1 expression in gastric cancers (*P*<0.001). Both EIF5A2 and MTA1 overexpression were correlated with pT stage (*P*=0.018 and *P*=0.042), pN stage (*P*=0.037 and *P*=0.020) and lymphovascular invasion (*P*=0.016 and *P*=0.044). EIF5A2 or MTA1 overexpression was significantly associated with poor overall survival and disease-free survival (All *P*<0.05). Multivariate analyses identified EIF5A2 as an independent predictor for both overall survival (*P*=0.012) and disease-free survival (*P*=0.008) in gastric cancer patients. Our findings indicate that EIF5A2 upregulation plays an important oncogenic role in gastric cancer. EIF5A2 may represent a new predictor for poor survival and is a potential therapeutic target for gastric cancer.

## Introduction

Gastric cancer (GC) is a highly metastatic disease and one of the most lethal of the gastrointestinal malignancies.[1] Despite advances in surgical and cytotoxic therapies for GC, the current treatments available to patients with advanced GC are very limited.[2, 3] To develop new therapeutic strategies, it is crucial to elucidate the molecular mechanisms that promote the invasive and metastatic properties of GC cells.

Eukaryotic translation initiation factor 5A2 (EIF5A2) is located at human chromosome 3q26.2, and is a member of the *EIF5A* gene family.[4] Overexpression of *EIF5A2* mRNA in certain human cancer cells, in contrast to overexpression limited to human testis and parts of brain, suggests *EIF5A2* is a potential oncogene.[4] Previous studies found that EIF5A2 was overexpressed in many human cancers such as pancreatic ductal adenocarcinoma, ovarian cancer, hepatocellular cancer, lung cancer, colorectal cancer and melanoma, and was correlated to poor survival of cancer patients and /or cancer cell aggressiveness.[5–11] Recent studies have demonstrated that EIF5A2 has carcinogenic abilities through its activation of the EIF5A2-MTA1/C-MYC axis.[8] However, little information is available about EIF5A2 protein expression, its prognostic significance and potential oncogenic role in human GC.

Accordingly, we first investigated the expression of *EIF5A2* in human GC cell lines and its potential role in cell proliferation, migration and invasion. Next, we identified possible downstream target proteins to elucidate the impact of EIF5A2 depletion or upregulation on the cellular functions of GC cells. Finally, we analyzed the correlation of EIF5A2 and MTA1 expression in human GC and its relevance to clinicopathological factors and survival in GC patients.

## Materials and Methods

### Ethics Statement

The study was approved by the Ethics Committee of PUMCH, Chinese Academy of Medical Science and Peking Union Medical College, Beijing, China, and written informed consent was obtained from each patient.

### Patients and specimens

GC tissue and matched adjacent non-tumor tissue samples were obtained from 160 consecutive patients who underwent surgical resection for primary GC at Peking Union Medical College Hospital (PUMCH) between January 2002 and December 2006. No patients received neoadjuvant chemotherapy or radiotherapy. The survival data were obtained based on both the patients’ records and telephone follow-up. The median follow-up time was 53 months (range, 1–113 months). Another two pairs of fresh GC tissues and noncancerous gastric mucosa tissues were obtained from patients who underwent surgical resection for poorly differentiated adenocarcinoma of stomach at PUMCH in 2014.

We defined lymphovascular invasion as the presence of tumor cell emboli within spaces surrounded by a clearly visualized endothelial lining in the periphery of tumor sections.[12, 13] Patients were staged according to the 7th edition of the AJCC TNM classification for carcinoma of the stomach.[14] Lauren histotype was divided into intestinal and diffuse-mixed type categories.[15]

### Cell culture

Five types of human GC cell lines were obtained from the Cell Center of Shanghai Institutes for Biological Sciences (AGS and MGC803, Shanghai, China) and the Cell Center of Institute of Basic Medical Sciences (MKN45, SGC7901 and HGC27, Beijing, China). The immortalized gastric mucosal epithelial cell line GES-1 was obtained from Beijing ComWin Biotech Co., Ltd (Beijing, China). All cells were cultured in RPMI 1640 medium supplemented with 10% fetal bovine serum (FBS; Life Technologies, Carlsbad, CA, USA) at 37°C in a humidified air atmosphere containing 5% CO_2_. Cells in logarithmic growth phase were used for further experiments.

### Knockdown EIF5A2 or MTA1 by small-interfering RNAs (siRNAs)

The **s**iRNA specifically against EIF5A2 and MTA1[16] and their non-targeting control siRNA (Life Technologies, Carlsbad, CA, USA) were chemically synthesized for this study. The EIF5A2 siRNA sequences were as follows: #1: 5’-GGAUCUUAAACUGCCAGAATT-3’, 5’- UUCUGGCAGUUUAAGAUCCTT-3’; #2: 5’-GGUUCACCUUGUUGGAAUUTT-3’, 5’- AAUUCCAACAAGGUGAACCTT-3; and #3: 5’-GCUUCCAGCACUUACCCUATT-3’, 5’-UAGGGUAAGUGCUGGAAGCTT-3; the non-targeting control (NC1) siRNA: 5’-UUC UCC GAA CGU GUC ACG UTT-3’; 5’-ACG UGA CAC GUU CGG AGA ATT-3’). The MTA1 siRNA sequences (Target 2 siRNA, T2-siRNA) were: 5’-GCUGAGAGCAAGUUAAAGCdTdT-3’, 5’-GCUUUAACUUGCUCUCAGCdTdT-3’) and FAM-labeled siRNA (AM4620) was used as a non-targeting control siRNA (NC2).

Twenty-four hours after plating in six-well plates, GC cells were transfected with 100pmol EIF5A2-siRNA, MTA1-siRNA or control siRNA using Lipofectamine 2000 transfection reagent (Life Technologies, Carlsbad, CA, USA) according to the manufacturer’s instructions. The cells were harvested for western blotting at 48 h after transfection.

### Plasmid construction and transient transfection

EIF5A2 expression vector (PIRES2-EGFP-EIF5A2) was synthesized by Life Technologies. Cells were transfected with PIRES2-EGFP-EIF5A2 or the control plasmid using Lipofectamine 2000 (Life Technologies) according to the manufacturer’s instructions.

### Real-time quantitative RT-PCR

Total RNA was isolated using TRIzol Reagent (Life Technologies) according to the manufacturer’s protocol. PCR was performed using the sequences for EIF5A2: forward: 5’- AACTGCCAGAAGGTGAACTAGG-3’; reverse: 5’- GTTTCCGTTTATTTGCAGGGT-3’ and the sequences for MTA1: forward: 5’-CCGGGCCTGCGAGAGCTGTTACAC-3’; reverse: 5’-CACGGCTTCCAGCGGCTTGCGTAC-3’. PCR was performed using UltraSYBR Mixture (CWbio.Co.Ltd) according to the manufacturer’s protocol. Reverse transcription was performed using a PrimeScript RT Master Mix kit (TAKARA Biotechnology Co. Ltd., Dalian, China). The cDNA products were subjected to real-time PCR using a SYBR Premix Ex Taq II kit (TAKARA Biotechnology Co. Ltd.). Real-time PCR was performed in a StepOnePlus Real-Time PCR System (Applied Biosystems, Carlsbad, CA) using the following program: 95°C for 10 minutes, followed by 40 cycles of 95°C for 15 seconds, and 60°C for 1 minute. *GAPDH* mRNA in each sample was quantified as an endogenous control.

### Western blotting

Protein concentration was quantified using a BCA protein assay kit (Thermo Scientific Pierce). Sodium dodecyl sulfate polyacrylamide gel electrophoresis (SDS-PAGE) was used to separate cell lysates. Proteins were transferred to PVDF membranes and blocked with tris-buffered saline and 0.1% Tween 20 (TBST) containing 5% bovine serum albumin, and then incubated with the following primary antibodies at 4°C overnight: rabbit anti-EIF5A2 or -C-MYC (1:1000; Epitomics, USA, Catalog # 5549–1 or 1472–1), rabbit anti-MTA1,-E-cadherin or -vimentin (1:1000; Cell Signaling, USA, Catalog # 5647, 3195 or 5741), or mouse anti-GAPDH (1:1000; Santa Cruz, Catalog # sc-25778). The membranes were washed three times with TBST and incubated with horseradish peroxidase (HRP)-conjugated anti-rabbit or anti-mouse secondary antibodies (1:3000; Santa Cruz, USA, Catalog# sc-2004 or sc-2005) for 1 h at room temperature. The protein bands were visualized using ECL detection reagents (Pierce, USA). The relative protein expression levels were normalized to GAPDH.

### Cell proliferation assay

The proliferation of HGC27 and MKN45 cells was examined using a Cell Counting Kit-8 (CCK-8) (Dojindo, Japan) according to the manufacturer’s instructions. Briefly, 24 h after transfection with siRNA or plasmid, the cells were seeded at a density of 1×10^4^ cells/well in 96-well plates and cultured for 0, 24, 48 and 72 h. Then, at the different time points, 10μL CCK-8 solution was added to each well and incubated for 2 h. The absorbance was read at 450nm on a plate reader.

### Transwell assays

The migratory and invasive ability of GC cells was detected using 24-well transwell cell culture chambers (8.0μm pore size, Costar, Cambridge, MA, USA) according to the manufacturer’s instructions. For the cell invasion assay, the insert membranes were pre-coated with Matrigel (BD, San Diego, CA, USA). For the cell migration assay, no Matrigel was used. At 24 h post-transfection, the cells were seeded at an appropriate density (HGC27, 5×10^5^/ml; MKN45, 1×10^7^/ml) in the upper chamber and cultured in OPTI-MEM (GIBCO, USA) without FBS for a further 24 h. The cells were allowed to migrate towards the RPMI 1640 medium containing 20% FBS in the bottom chamber. The non-migratory cells on the upper membrane surface were removed with a cotton tip, and the migratory cells attached to the lower membrane surface were fixed with 4% paraformaldehyde and stained with H&E. The numbers of invaded cells were counted in five randomly selected fields under a microscope.

### Immunohistochemistry (IHC) staining and assessment

IHC staining was performed on 5μm-thick sections from paraffin-embedded specimens. Monoclonal rabbit anti-human EIF5A2 antibody (Epitomics, USA, Catalog # 5549–1), rabbit anti-human MTA1 antibody (Cell Signaling, USA, Catalog # 5647) and PV-6000 Polymer Detection System (ZSBG-BIO, China) were used for staining in accordance with the manufacturer’s instructions. In brief, slides with paraffin sections were deparaffinized with xylene and rehydrated with ethanol. Endogenous peroxidase activity was blocked with 3% hydrogen peroxide for 10 min. For antigen retrieval, slides were microwave-treated and boiled in a 0.01M citrate buffer (pH 6.0) for 10 min, and thereafter incubated with 0.1% trypsin at 37°C for 5 min. The slides were incubated with monoclonal rabbit anti-human EIF5A2 or MTA1 antibody (1:100 dilution) for 1.5 h at 37°C in a humidified chamber. As a negative staining control, primary antibody was replaced with non-specific rabbit IgG. Two independent pathologists evaluated the immunohistochemical staining of EIF5A2 and MTA1. A semiquantitative scoring method was used with reference to the previous study.[7]

### Statistical analysis

All analyses were performed using SPSS 12.0 software (Chicago, IL, USA). The McNemar test was used for comparison of EIF5A2 or MTA1 staining in human GC and adjacent non-tumor specimens. The chi-square test was used to compare differences between categorical variables, whereas the paired, two-tailed Student’s *t*-tests were used to compare differences between categorical variables. The correlation between EIF5A2 and MTA1 was analyzed using the Spearman’s rank test. For univariate survival analysis, survival curves were constructed using the Kaplan-Meier method and compared by the Log-rank test. The Cox proportional hazards model with multivariate analyses was used to investigate the independent prognostic factors. All *P* values were two-sided, and *P* values of <0.05 were considered to be statistically significant. All experiments were performed at least in technical and biological triplicates.

## Results

### EIF5A2 Expression in GC cells and tissues and validation of the intervention

**Fig 1:**
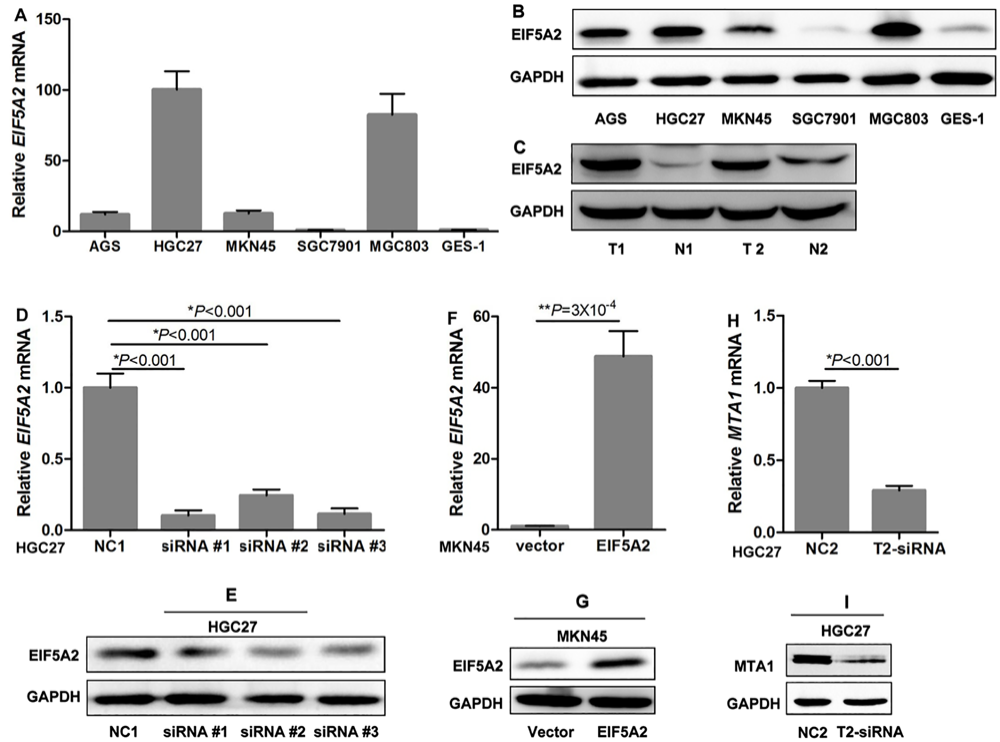
EIF5A2 Expression in GC cells and tissues and validation of the intervention. (**A**) *EIF5A2* mRNA expression was examined by real-time quantitative RT-PCR with *GAPDH* serving as a loading control. (**B**) Protein expression in cell lines was confirmed by western blot analysis with GAPDH as a loading control. (**C**) EIF5A2 protein in human GC tissues was higher than that of the paired distant non-tumor tissues. (D) All three EIF5A2-siRNAs, especially #1, efficiently repressed *EIF5A2* mRNA expression in HGC27 cells. (E) Western blotting reveals that EIF5A2 was knocked down by the treatment of EIF5A2- siRNAs in HGC27 cells. (**F**) *EIF5A2* mRNA was significantly upregulated by transient transfection of EIF5A2 plasmids in MKN45 cells. (**G**) EIF5A2 protein was significantly upregulated by transient transfection of EIF5A2 plasmids in MKN45 cells. (H and I) T2-siRNA could efficiently suppress MTA1 expression in HGC27 cells.

We investigated the expression of EIF5A2 in five GC cell lines and the immortalized gastric mucosal epithelial cell line GES-1 *in vitro* by both qRT-PCR (Fig. 1A) and western blotting (Fig. 1B). EIF5A2 protein in human GC tissues was higher than that of the paired distant non-tumor tissues (Fig. 1C). Because of the high endogenous expression level of EIF5A2 and the relatively high transfection efficiency in HGC27, we selected this cell line for RNAi analysis, and selected MKN45 cells for EIF5A2 upregulation experiments due to its relatively low EIF5A2 expression and high transfection efficiency.

All three siRNAs could efficiently suppress EIF5A2 expression in HGC27 cells (Fig. 1D, E). We used siRNA#1 as EIF5A2-targeted siRNA (T1-siRNA) in the subsequent experiments. EIF5A2 expression was significantly upregulated by transient transfection of EIF5A2 plasmids in MKN45 cells (Fig. 1F, G). T2-siRNA could efficiently suppress MTA1 expression in HGC27 cells (Fig. 1H, I).

### EIF5A2 or MTA1 expression levels influenced the aggressiveness of GC cells in vitro

**Fig 2:**
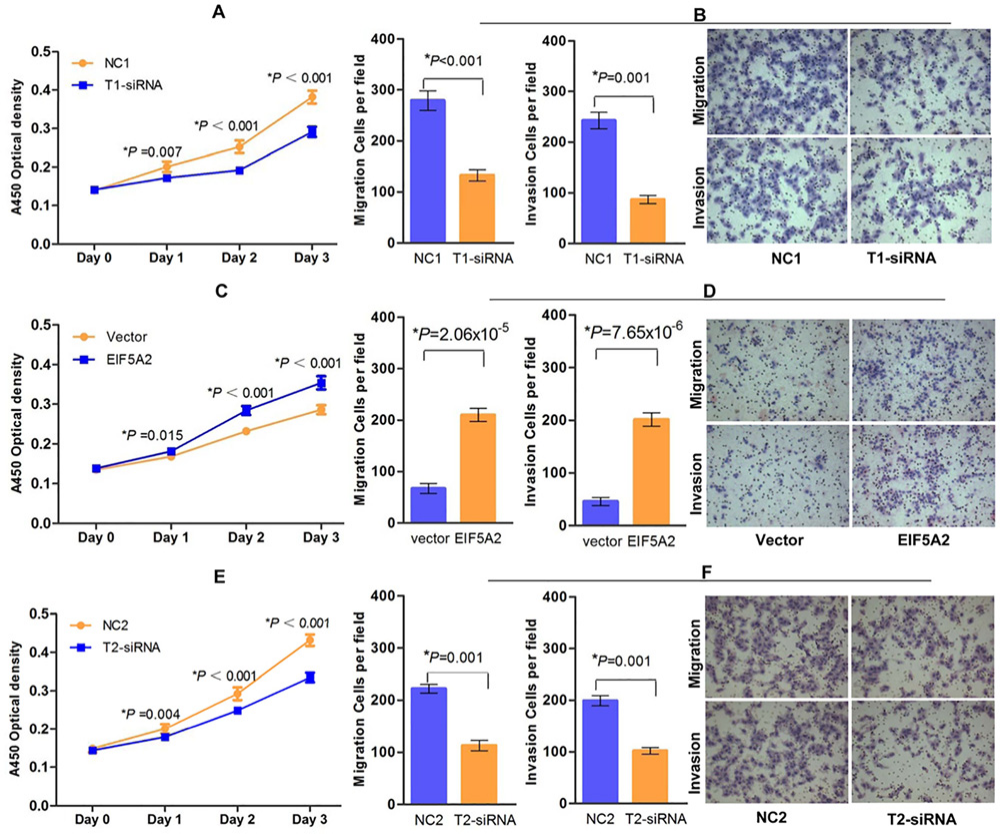
EIF5A2 or MTA1 expression levels influenced the aggressiveness of GC cells in vitro. (**A**) Proliferation of EIF5A2-targeted siRNA (T1-siRNA) and non-targeted control (NC) HGC27 cells were measured by CCK-8 assay every 24 h after siRNA transfection. (**B**) Cell migration and invasion were decreased following transfection of T1-siRNA into HGC27 cells (Student’s *t*-test, *P*<0.001 and *P* = 0.001, respectively). Column, mean; bars, ±SD (from triplicates). (**C**) Proliferation of EIF5A2-plasmid and control plasmid MKN45 cells were measured by CCK-8 assay every 24 h after plasmid transfection. (**D**) Cell migration and invasion were increased following transient transfection of EIF5A2-plasmid into MKN45 cells (Student’s *t*-test, *P* = 2.06×10^-5^ and *P* = 7.65×10^-6^, respectively). Column, mean; bars, ±SD (from triplicates). Image insert (B right and D right) shows a representative field of view from each treatment. (**E**) Proliferation of MTA1-targeted siRNA (T2-siRNA) and non-targeted control (NC2) HGC27 cells were measured by CCK-8 assay every 24 h after siRNA transfection. (**F**) Cell migration and invasion were decreased following transfection of T2-siRNA into HGC27 cells (Student’s *t*-test, both *P* = 0.001). Column, mean; bars, ±SD (from triplicates).

Knockdown of EIF5A2 by T1-siRNA significantly inhibited cell proliferation (Fig. 2A), migration and invasion (Fig. 2B) in HGC27 cells compared with those of the parental cells. Upregulation of EIF5A2 by EIF5A2 plasmid expression promoted increased proliferation (Fig. 2C), migration and invasion (Fig. 2D) of MKN45 cells. Knockdown of MTA1 by T2-siRNA significantly inhibited cell proliferation (Fig. 2E), migration and invasion (Fig. 2F) in HGC27 cells compared with those of the parental cells.

### Possible target gene expression after EIF5A2 knockdown or overexpression

**Fig 3:**
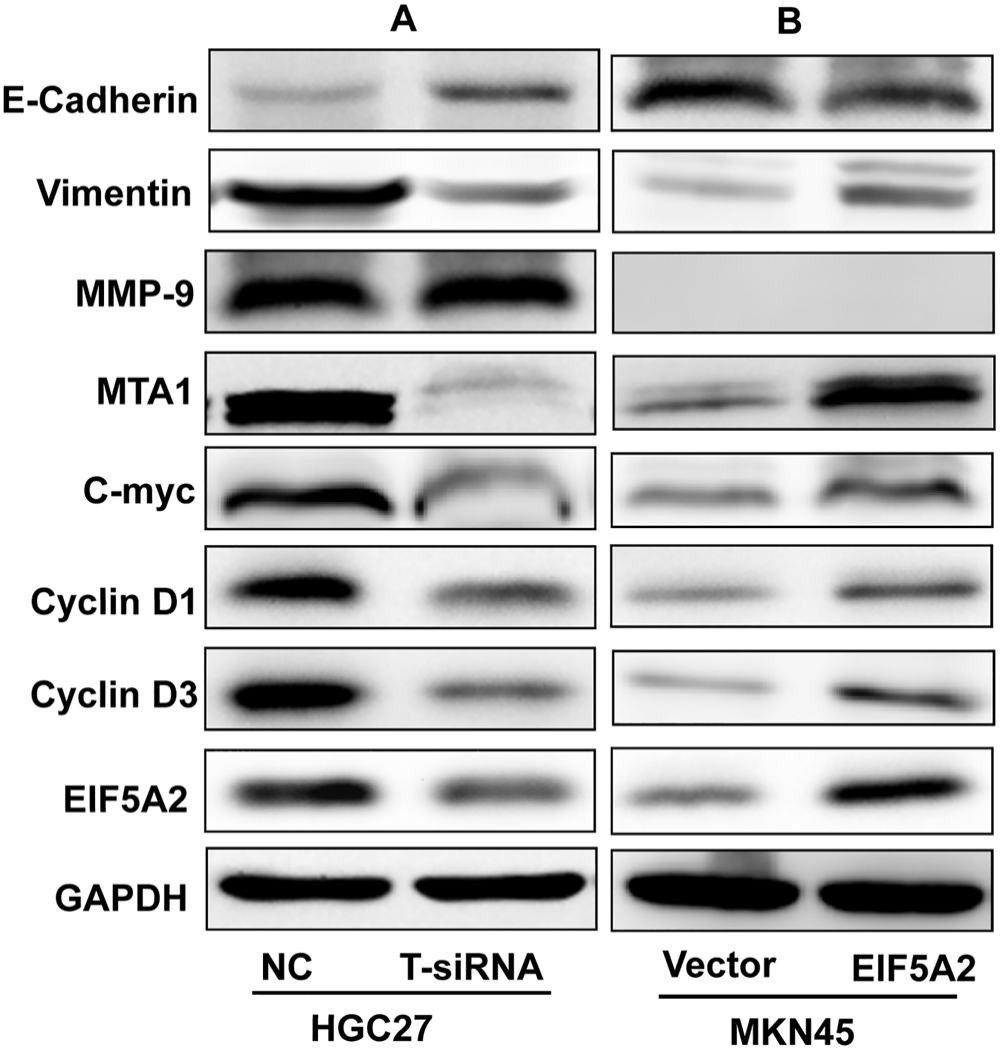
Immunoblotting analysis of protein expression after silencing or overexpressing EIF5A2 in GC cells. (**A**) After knockdown of EIF5A2 in HGC27 cells, E-cadherin levels were upregulated, while vimentin was downregulated accompanied by cyclin D1, cyclin D3 and C-MYC, and MTA1 downregulation. (**B**) Ectopic overexpression of EIF5A2 after transient transfection of EIF5A2-plasmid substantially upregulated cyclin D1, cyclin D3, C-MYC and MTA1, accompanied by downregulation of E-cadherin, while vimentin was upregulated.

After knockdown of EIF5A2 in HGC27 cells, the levels of the epithelial marker E-cadherin were upregulated, while the levels of mesenchymal marker vimentin were downregulated and accompanied by proliferation-related proteins cyclin D1 and cyclin D3 and metastasis-associated C-MYC and MTA1 downregulation (Fig. 3A). In contrast, after EIF5A2 overexpression in MKN45 cells, the expression of E-cadherin was downregulated, while the level of vimentin was upregulated and accompanied by cyclin D1, cyclin D3, C-MYC and MTA1 upregulation (Fig. 3B). Knockdown or overexpression of EIF5A2 had no significant effect on MMP9 expression in HGC27 and MKN45 cells (Fig. 3A, B).

### Association of EIF5A2 and MTA1 expression with clinicopathological features in patients with GC

**Fig 4:**
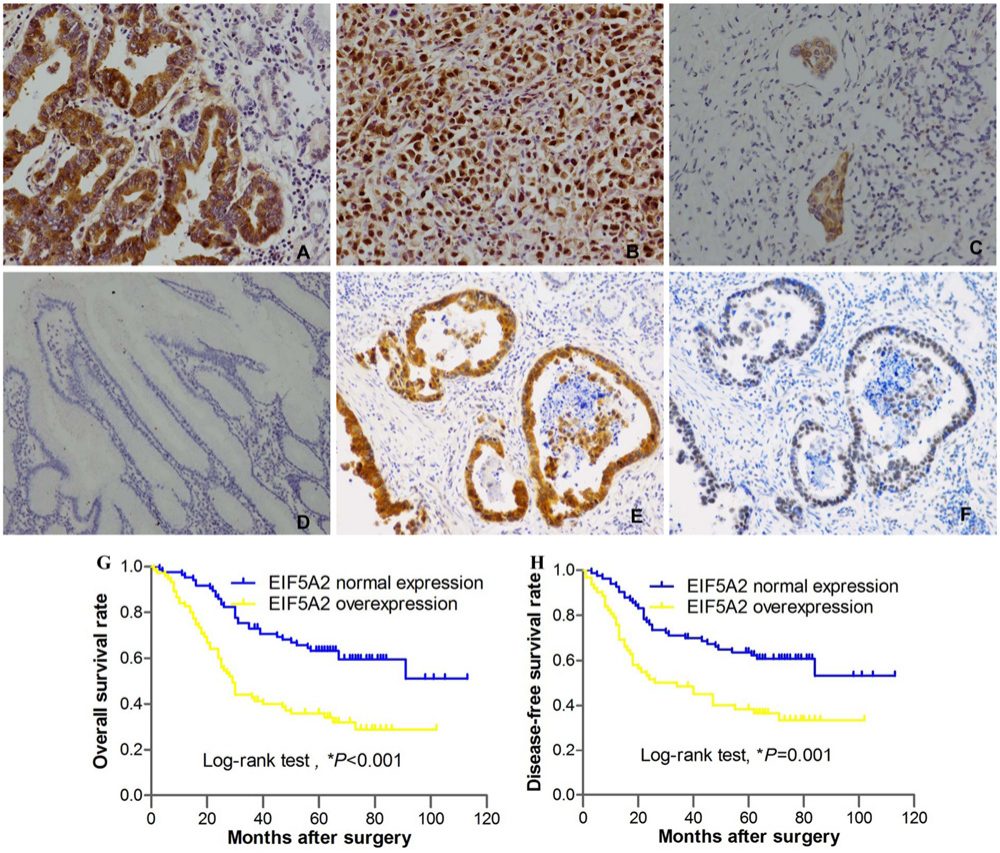
EIF5A2 Expression in human GC tissues and its prognostic significance. (**A**) Representative immunohistochemistry images showing EIF5A2 overexpression in moderately-differentiated adenocarcinoma (×200). (**B**) Overexpression of EIF5A2 in poorly-differentiated gastric adenocarcinoma (×200). (**C**) Overexpression of EIF5A2 in tumor cells invading vessels (×200). (**D**) Normal expression of EIF5A2 in non-tumor tissue (×100). (**E**) The typical staining images showing both EIF5A2 and MTA1 overexpression in the same gastric adenocarcinoma; (**G**) Overall survival curves for 160 gastric cancer patients receiving gastrectomy, grouped according to EIF5A2 expression (*P*<0.001). (**H**) Disease-free survival curves for 145 gastric cancer patients receiving curative surgery, grouped according to EIF5A2 expression (*P* = 0.001).

Positive signals of EIF5A2 were predominantly located in the cytoplasm and nucleus of tumor cells, while MTA1 signals were mainly localized in the nucleus of tumor cells (Fig. 4). We defined the immunostaining values of 0–3 as the normal expression level of EIF5A2 or MTA1, while immunostaining values of 4–12 was defined as overexpression of EIF5A2 or MTA1. EIF5A2 overexpression was found in 75 of the tumor specimens, compared with only seven of 160 matched adjacent non-tumor mucosal tissues (*P*<0.001). Representative IHC staining of EIF5A2 in moderately- or poorly-differentiated gastric adenocarcinoma, vessel invasion tumor cells and non-tumor tissue is shown in Fig. 4A, B, C and D, respectively. Of the 160 cases analyzed, 70 (43.75%) were positive for MTA1, Statistical analysis of the expression patterns showed that there was a positive correlation between EIF5A2 and MTA1 expression (r = 0.510, *P*<0.001). The typical staining images for overexpression of EIF5A2 and MTA1 in gastric adenocarcinoma were shown in Fig. 4E and 4F.

As shown in Table 1, both EIF5A2 and MTA1 overexpression were positively correlated with more advanced pT stage (*P* = 0.018 and *P* = 0.042), pN stage (*P* = 0.037 and *P* = 0.020) and positive lymphovascular invasion (*P* = 0.016 and *P* = 0.044), whereas they were not associated with other clinicopathological features.

**Table 1:** Association between EIF5A2 or MTA1 expression and clinicopathological characteristics.

Variables	No. of cases	EIF5A2 expression		*P* value	MTA1 expression		*P* value
		Normal	Over		Normal	Over	
Gender				0.615			0.311
Women	46	23	23		23	23	
Men	114	62	52		67	47	
Age (years)				0.518			0.745
≤65	96	49	47		53	43	
>65	64	36	28		37	27	
Lauren histotype				0.652			0.586
Intestinal	67	37	30		36	31	
Diffuse-mixed	93	48	45		54	39	
Tumor size (cm)				0.293			0.814
≤5.0	109	61	48		62	47	
>5.0	51	24	27		28	23	
Tumor location				0.726			0.675
Upper third	36	18	18		18	18	
Middle third	25	12	13		15	10	
Low third	99	55	44		57	42	
LVI				0.016			0.044
Present	57	23	34		26	31	
Absent	103	62	41		64	39	
pT stage				0.018			0.042
T1	21	16	5		16	5	
T2	28	19	9		19	9	
T3	16	8	8		10	6	
T4	95	42	53		45	50	
pN stage				0.037			0.020
N0	43	29	14		32	11	
N1	38	23	15		22	16	
N2	43	18	25		21	22	
N3	36	15	21		15	21	

EIF5A2, eukaryotic translation initiation factor 5A2; LVI, lymphovascular invasion; MTA1, Metastasis-associated protein 1.

### Increased EIF5A2 or MTA1 expression correlated with poorer survival in GC patients

At the last follow-up, 75 of 160 patients had died. Of these, 73 people died of tumor recurrence. The Kaplan-Meier analysis showed that both the 5-year overall survival (OS) and disease-free survival (DFS) for the EIF5A2 overexpression group were shorter than those for the normal EIF5A2 expression group (*P*<0.001 and *P* = 0.001, Fig. 4G, H). Consistently, patients with MTA1 overexpression displayed a poor prognosis for OS and DFS (*P*<0.001 and *P* = 0.001).

Variables with significance in univariate analysis by the Kaplan-Meier methods were included in the multivariate Cox regression analyses. As shown in S1 Table and S2 Table, multivariate analysis identified that overexpression of EIF5A2 was an independent factor influencing both OS (hazard ratio 1.831, 95%CI 1.135 to 2.857, *P* = 0.012, S1 Table) and DFS (hazard ratio 1.880, 95%CI1.177 to 3.002, *P* = 0.008, S2 Table) of patients receiving curative resection for GC. However, the overexpression of MTA1 did not produce independent prognostic significance for OS and DFS in the multivariate analysis.

## Discussion

Eukaryotic initiation factor 5A 2 (EIF5A2) is localized at a chromosomal region often noted for chromosomal instability in GC and many other cancers, 3q.[17–20] EIF5A2, as one of the only two isoforms of EIF5A family, is confirmed as an oncogenic protein and may also be an important biomarker for the prognosis and therapeutic target of many kinds of human tumors.[21] Although a previous study had examined *EIF5A2* gene expression in primary GC, the current study provides the first evidence of the clinical significance of EIF5A2 expression in human GC and its possible role in the regulation of GC cell aggressiveness.[22]

We first performed EIF5A2 knockdown and overexpression experiments *in vitro*. The results showed that suppression of EIF5A2 in HGC27 cells led to significant decreases in cell proliferation, migration and invasion, while its upregulation in MKN45 cells resulted in the converse. These results are consistent with previous findings in other tumors.[8, 10, 23] We also found that MTA1 knockdown in HGC27 cells significantly inhibited cell proliferation, migration and invasion. But the mechanism by which EIF5A2 enhances GC cell aggressiveness is still unknown.

Therefore, we performed further studies of the possible targets of EIF5A2 *in vitro*. Our results showed that EIF5A2 positively regulated cyclin D1 and cyclin D3, which play important roles in GC cell proliferation and cell cycle regulation.[24, 25] All these results supported the hypothesis that EIF5A2 promotes GC cell proliferation via upregulation of cyclin D1 and cyclin D3. At the same time, previous studies showed that EIF5A2 promotes cell invasion/metastasis and epithelial-mesenchymal transition (EMT) through activating MTA1/C-MYC in colorectal cancer.[8] MTA1 and C-MYC, as well as the EMT program, were also involved in regulation of GC metastasis.[26–28] We therefore examined MTA1, C-MYC and two EMT-associated markers, E-cadherin and vimentin. Our studies demonstrated that EIF5A2 positively regulated MTA1, C-MYC and vimentin and negatively regulated E-cadherin. All these results suggest that EIF5A2 might regulate cell migration and invasion by regulation of MTA1, C-MYC and EMT *in vitro*, but further studies are required to investigate the exact mechanisms.

The results of the current study also showed that EIF5A2 protein was positively correlated with pT stage and pN stage. Similar results were also observed in human ovarian cancer.[5] These results suggest that EIF5A2 may be associated with tumor invasion and lymph node metastasis in certain types of human cancers, including GC. More interestingly, in the current study, EIF5A2 overexpression was also found in invading cancer cells (vessel invasion), and overexpression of EIF5A2 protein in primary GC tissue correlated with the presence of lymphovascular invasion. In accordance with the results found in previous studies of other human malignancies including epithelial ovarian tumors, bladder carcinoma, lung cancer, and colorectal cancer, overexpression of EIF5A2 in the current study was correlated with poor survival of patients with GC.[5, 8, 29] In addition, multivariate analysis showed that EIF5A2 protein is an independent predictor for poor survival in patients undergoing surgery for GC.

MTA1, as an candidate metastasis-associated gene, have been demonstrated to play the critical role in gastric cancer cell aggressiveness.[27] Overexpression of MTA1 is significantly associated with poor prognosis in pN0 GC patients.[30] The current study showed that EIF5A2 expression was positively correlated with MTA1 expression in human GC tissues. Immunohistochemical analysis combined in vitro studies shown that the EIF5A2-MTA1–axis may play an important role in gastric cancer aggressiveness.

In summary, the results of the current study showed that the overexpression of EIF5A2 was closely related to GC cell proliferation, migration and invasion via metastasis-associated proteins MTA1. At the same time, EIF5A2 may be important in EMT regulation in GC. Overexpression of EIF5A2 could be an independent factor predicting poor survival and an attractive therapeutic target for GC, but further studies are required.

## Supporting Information

S1 Table Analysis of factors associated with overall survival (OS). (DOC)

S2 Table Analysis of factors associated with disease-free survival (DFS). (DOC)
