# Supplementary material for: Overexpression of Eukaryotic Translation Initiation Factor 5A2 (EIF5A2) Correlates with Cell Aggressiveness and Poor Survival in Gastric Cancer
Source: PLoS One. 2015 Mar 20;10(3):e0119229. doi: 10.1371/journal.pone.0119229 (PMC4368542; doi:10.1371/journal.pone.0119229)
Supplement: S1 Table — (DOC) [file pone.0119229.s001.doc]

S1_Table. Univariate and multivariate analysis of factors associated with overall survival (OS).

| Variables | n | Univariate analysis^a^ | | Multivariate analysis^b^ | |
| --- | --- | --- | --- | --- | --- |
|  |  | 5-y OS | *P* value | Hazard ratio (95%CI) | *P* value |
| Gender |  |  | 0.454 |  |  |
| Women | 46 | 56.1 |  |  |  |
| Men | 114 | 48.0 |  |  |  |
| Age (years) |  |  | 0.195 |  |  |
| ≤65 | 96 | 54.7 |  |  |  |
| >65 | 64 | 43.8 |  |  |  |
| Lauren histotype |  |  | 0.376 |  |  |
| Intestinal | 67 | 56.1 |  |  |  |
| Diffuse-mixed | 93 | 45.6 |  |  |  |
| Tumor size (cm) |  |  | <0.001 |  |  |
| ≤5.0 | 109 | 59.5 |  |  |  |
| >5.0 | 51 | 24.4 |  |  |  |
| Tumor location |  |  | <0.001 |  | 0.008 |
| Low third | 99 | 61.2 |  | 1.0 |  |
| Upper or Middle third | 61 | 27.0 |  | 1.831 (1.169-2.867) |  |
| Lymphovascular invasion |  |  | <0.001 |  | 0.013 |
| Absent | 103 | 63.7 |  | 1.0 |  |
| Present | 57 | 26.3 |  | 1.769 (1.130-2.770) |  |
| pT stage |  |  | <0.001 |  |  |
| pT1-2 | 49 | 77.5 |  |  |  |
| pT3-4 | 111 | 38.2 |  |  |  |
| pN stage |  |  | <0.001 |  | <0.001 |
| N0 | 43 | 90.5 |  | 1.0 |  |
| N1-3 | 117 | 35.5 |  | 4.641(2.085-10.332) |  |
| Radical extent |  |  | <0.001 |  | <0.001 |
| R0 | 145 | 55.6 |  | 1.0 |  |
| R1, R2 | 15 | 0.0 |  | 3.203 (1.748-5.869) |  |
| EIF5A2 expression |  |  | <0.001 |  | 0.012 |
| Normal | 85 | 63.2 |  | 1.0 |  |
| Over | 75 | 35.8 |  | 1.831 (1.135-2.857) |  |
| MTA1 expression |  |  | <0.001 |  |  |
| Normal | 90 | 61.7 |  |  |  |
| Over | 70 | 35.7 |  |  |  |

^a^Log-rank test; ^b^Cox proportional hazards model.
