# Supplementary material for: Overexpression of Eukaryotic Translation Initiation Factor 5A2 (EIF5A2) Correlates with Cell Aggressiveness and Poor Survival in Gastric Cancer
Source: PLoS One. 2015 Mar 20;10(3):e0119229. doi: 10.1371/journal.pone.0119229 (PMC4368542; doi:10.1371/journal.pone.0119229)
Supplement: S2 Table — (DOC) [file pone.0119229.s002.doc]

S2_Table. Univariate and multivariate analysis of factors associated with disease-free survival (DFS)

|  | Univariate analysis^a^ | | | Multivariate analysis^b^ | |
| --- | --- | --- | --- | --- | --- |
| Variables | n | 5-y DFS | *P* value | Hazard ratio (95%CI) | *P* value |
| Gender |  |  | 0.699 |  |  |
| Women | 42 | 56.8 |  |  |  |
| Men | 103 | 51.2 |  |  |  |
| Age (years) |  |  | 0.473 |  |  |
| ≤65 | 90 | 56.3 |  |  |  |
| >65 | 55 | 47.3 |  |  |  |
| Lauren histotype |  |  | 0.486 |  |  |
| Intestinal | 62 | 58.1 |  |  |  |
| Diffuse-mixed | 83 | 48.8 |  |  |  |
| Tumor size (cm) |  |  | <0.001 |  |  |
| ≤5.0 | 103 | 61.9 |  |  |  |
| >5.0 | 42 | 30.4 |  |  |  |
| Tumor location |  |  | <0.001 |  | 0.011 |
| Low third | 92 | 64.0 |  | 1.0 |  |
| Upper or Middle third | 53 | 32.9 |  | 1.854 (1.153-2.980) |  |
| Lymphovascular invasion |  |  | <0.001 |  | 0.023 |
| Absent | 96 | 65.3 |  | 1.0 |  |
| Present | 49 | 28.6 |  | 1.754 (1.080-2.847) |  |
| pT stage |  |  | <0.001 |  |  |
| pT1-2 | 49 | 75.5 |  |  |  |
| pT3-4 | 96 | 41.0 |  |  |  |
| pN stage |  |  | <0.001 |  | <0.001 |
| N0 | 43 | 88.3 |  | 1.0 |  |
| N1-3 | 102 | 37.9 |  | 4.657 (2.098-10.334) |  |
| EIF5A2 expression |  |  | 0.001 |  | 0.008 |
| Normal | 83 | 63.6 |  | 1.0 |  |
| Over | 62 | 36.5 |  | 1.880 (1.177-3.002) |  |
| MTA1 expression |  |  | 0.001 |  |  |
| Normal | 84 | 63.9 |  |  |  |
| Overexpression | 61 | 37.7 |  |  |  |

^a^Log-rank test; ^b^Cox proportional hazards model.
